# A Critical Review of the Literature Regarding the Selection of Long-Term Romantic Partners

**DOI:** 10.1007/s10508-023-02646-y

**Published:** 2023-07-07

**Authors:** Scott Devenport, Catriona Davis-McCabe, Sam Winter

**Affiliations:** School of Population Health, Curtin University, Bentley, WA 6102 Australia

**Keywords:** Mate selection, Trait preference, Evolutionary psychology, Romantic relationships, Romantic selection, Intimate relationships

## Abstract

Research regarding how people choose their long-term romantic partners is extensive, but the understanding of the psychological processes behind these choices, and predicting who people choose, is elusive. This review attempts to examine potential reasons for this elusive nature by first outlining the current state of the literature and then highlighting issues within the current paradigm. First among these issues is a focus on singular perspectives and little attempt to integrate these perspectives with others. Second, many studies focus on increasingly complex designs to explore the predictive utility of trait preferences, attempts which have had only limited success. Third, novel findings appear to be unintegrated with established findings, leaving the potential combination of these ideas unrealized. Finally, long-term romantic partner selection is a complex psychological phenomenon, but current theory and research methodologies are not sufficiently addressing this complexity. This review concludes with suggestions for future research direction, including a focus on the psychology behind the partner selection process and the potential of qualitative enquiry to reveal novel pathways behind these psychological processes. There is a need for an integrative framework that permits the coexistence of established and novel ideas, and multiple perspectives, from both current and future research paradigms.

## Introduction

Selecting a long-term romantic partner is an important experience that is nearly universal across human societies and cultures (Buss, 1989; Marlowe, 2004). This process takes many forms across societies and cultures (for example, arranged marriage; Banerjee et al., 2013), but most research has focused on the Western concept of personal choice in partner selection (Fletcher et al., 2019). In Western settings, selecting a suitable partner has been linked to improved relationship quality (Conroy-Beam et al., 2016; Valentine et al., 2019), which in turn is linked to generally improved health (Robles et al., 2014). The importance of the partner selection process has inspired nearly a century of academic enquiry (Eastwick et al., 2022; Hill, 1945), which has revealed a complex psychological phenomenon (Sparks et al., 2020). Attempts to address this complexity in research continue; however, recent sound efforts have had limited success (Eastwick et al., 2022; Joel et al., 2020).

Throughout this critical review, the case is made that, despite innovation, the field of partner selection research appears resistant to integration of perspectives and is methodologically inflexible. The review describes and critiques mostly Western literature that concerns the partner selection process of individuals who choose long-term romantic partners. First, theories that have been developed to explain the partner selection process will be addressed. Second, methodologies and findings employing these theories will be discussed. Third, ideas and studies that attempt to address challenges in the field are detailed. Lastly, outstanding issues in the field are outlined before suggestions are made so researchers can begin to solve these problems and address the complexity of the partner selection process.

## Theories of the Partner Selection Process

### Evolutionary Theory

Numerous theories have been applied to the partner selection process, but none more so than evolutionary theory (Griskevicius et al., 2015; Li & Meltzer, 2015). The theory originally proposed that evolutionary pressure acts upon organisms to reproduce successfully (Darwin, 1871). The way that the theory has been applied subsequently within partner selection literature suggests that people primarily select partners based on their perception of a potential partner’s reproductive fitness (Finkel & Eastwick, 2015). A potential partner’s reproductive fitness might be subjectively judged based on numerous traits, from healthy appearance and symmetry (Perrett et al., 1999) to fidelity or chastity (Buss, 1989), and potential parenting suitability (Finkel & Eastwick, 2015).

It is important to highlight the diverse ways that the term “reproductive fitness” is used in the literature. When the term is used in reference to partner selection, it can encapsulate many features; one reason for this is that individuals cannot observe reproductive fitness directly and can only form a model of it based on observations or information of other features (Symons, 1992). Individuals’ models of reproductive fitness are various, further supporting the notion that it cannot be observed directly (Fletcher et al., 2004). Arguably, the nature of reproductive fitness is simplified in the literature and can appear to suggest reproduction is at the forefront of choice (Conroy-Beam et al., 2019), a conclusion that is likely to be incorrect (Symons, 1992). The literature so frequently utilises this simplification that it is difficult to discuss the field in other terms. Therefore, we use the simplified term throughout this review but also attempt to speak to the diversity of the term in our critique.

Evolutionary perspectives frequently focus on sex differences, the core evolutionary idea of differences between biological males and females (Eastwick & Finkel, 2008; Regan et al., 2000; Schmitt, 2003). Trivers’ (1972) parental investment theory is an influential example of this focus on sex difference. Trivers suggests that women are likely to be more selective of partners in short-term contexts than men, due to the risks of becoming pregnant. In long-term contexts however, there should be minor differences between sexes as the parties are equally invested. In both short- and long-term contexts, the theory has been supported by research (Buss & Schmitt, 1993; Kenrick et al., 1990).

Consistent sex differences arguably provide evidence for the evolutionary underpinnings of partner selection and so do cross-cultural comparisons as exemplified by Buss (1989). Buss found strong consistencies in sex differentiated preferences across the 37 cultures studied (37 countries/ethnic groups). Men generally preferred physical attractiveness and younger partners more than women. Women still rated physical attractiveness highly, though not as highly as men, and preferred older partners. Less convincing for evolutionary theory was preference for chastity in a partner, where fewer cultures had significant differences between sexes, perhaps due to more progressive ideals (Wood & Eagly, 2002). Buss’ study was a landmark in support for evolutionary psychological theory regarding partner selection and continues to be influential in the field (Buss et al., 2020; Thomas et al., 2019).

Notwithstanding support in the literature, there may be a need for a more critical view of evolutionary theory within the partner selection field (Ross & Hall, 2020; Wood & Eagly, 2002). The evolutionary perspective on partner selection is heavily focused on reproduction and this focus may not entirely suit contemporary reality. For example, at least 7–8% of Australian adults have reported not wanting to have children, presumably making reproductive fitness a less crucial factor for these individuals’ partner selection choices (Weston et al., 2005). Second, reproduction is a heterosexual-exclusive function. People of other sexual identities may privilege other factors beyond reproductive fitness and this needs to be considered (van Anders, 2015), particularly in relation to societies where same-sex attracted people consider themselves free to operate in romantic spaces (Rostosky & Riggle, 2017). Lastly, an overly heavy emphasis on an evolutionary perspective can potentially blind researchers and others to the impacts of other social and cultural constructs, such as familial influence and gender equality, which appear to be important in some partner selection contexts (Bejanyan et al., 2015; Wood & Eagly, 2002).

Evolutionary mechanisms arguably influence individual decisions in partner selection, even if an individual does not want to reproduce and cannot perceive reproductive fitness (van Anders, 2015). This is the nature of evolved adaptations which influence an individual even if the adaptation is not related to reproduction or partner selection (Symons, 1992). Evolutionary perspectives add vital information to this field of research, and this should not be overlooked. However, there appears to be a need to look elsewhere to account for cultural changes and further our overall understanding of partner selection phenomena (Ross & Hall, 2020).

### Social Psychological Theory

Long-term romantic relationships are interpersonal endeavours and therefore social in nature (Finkel & Eastwick, 2015). Accordingly, researchers have reflected upon about how partners are selected from a social psychological perspective. Two examples of potential social influences on partner selection are familiarity and similarity (Finkel & Eastwick, 2015). Regarding familiarity, it is suggested that as someone becomes more familiar with a potential partner, attraction increases and there is a higher desire for a relationship (Reis et al., 2011). This idea is supported in the literature (Back et al., 2008), but appears to be complicated, and other concurrent factors impacting partner selection may need to be considered (Eastwick et al., 2023). With similarity, the common proposition is that “likes attract,” the more similar two people are, the more likely it is that they will be attracted to one another (Finkel & Eastwick, 2015). The impact of similarity on partner selection appears complicated (Finkel & Eastwick, 2015; Sprecher et al., 2015). For example, a meta-analytic review conducted by Montoya et al. (2008) concluded that it was not how similar two people were, but how similar the two perceived they were that drove attraction. A study by Cerasa et al. (2022) describes the complexities in this area. They found that more neurotic men tended to end up with more extraverted women, which the Cerasa et al. state supports the idea that “opposites attract.” However, the same study also found support for the “likes attract” idea, making it unclear which is more useful in explaining this area of partner selection.

Thibaut and Kelley’s (1959) interdependence theory has been widely applied to intimate relationships and romantic partner selection (Campbell & Fletcher, 2015; Fletcher et al., 1999). Interdependence theory frames partner selection in terms of the rewards that an individual can gain from a potential or current partner and rewards available from alternatives. Individuals compare what they think they deserve to what they are receiving, or could receive, from a potential partner. If that individual believes that they could not receive what they think they deserve, they may consider what they could gain from alternatives. In terms of partner selection, interdependence theory has been applied mostly in the terms of ideal standards: people generate a set of ideals regarding potential partners based on what they desire, what they think they deserve, and what they think they can attain (Fletcher et al., 1999). Despite a large body of the literature, studies applying ideal standards in a variety of ways have had mixed results, perhaps indicating something is missing in the model (Conroy-Beam et al., 2022; Eastwick et al., 2018; Fletcher et al., 2020; Sparks et al., 2020).

In partner selection, social psychological and evolutionary perspectives are not easily integrated with each other (Conroy-Beam, 2021). Conroy-Beam highlights that each perspective cannot explain some of the core principles that are explained by the other. Finkel and Eastwick (2015) believe that a cause of this disconnect is the focus on rewards in each perspective. They suggest that instead of rewards, research should focus on goals, especially goals of high motivational priority. This approach would facilitate application of evolutionary theories in situations where goals are primarily around reproduction. Social psychological theories could be applied when goals are more interpersonal or driven by social constructs, such as cultural pressures. These integrative ideas are in their infancy but appear promising, even if some applications have yet to produce results (Eastwick et al., 2023).

### Other Theories and Models of Partner Selection

While evolutionary and social psychological perspectives are dominant in the field, numerous other theories of long-term romantic partner selection are available, each supported to some extent by bodies of research (Finkel & Eastwick, 2015; Sprecher et al., 2015). For example, researchers have drawn from the economic literature to develop “market forces” theories of partner selection (Buss & Barnes, 1986; Wood & Brumbaugh, 2009). Wood and Brumbaugh (2009) describe the “dating market,” in which potential partners have varying levels of desirable and undesirable traits. Desirability could be a function of individual preference or generalised norms identified in research, such as attractiveness and agreeableness. Within this perspective, a person with higher levels of desirable traits is more desired by others, increasing their “value” and thus, increasing the number of people interested in them—and consequently whom they can choose. The opposite plays out for a person with higher levels of undesirable traits; their lower value reduces the number of people interested in them and whom they can consequently choose as mutual interest is likely a necessary qualifier for relationship formation (Günaydin et al., 2013).

Wood and Brumbaugh (2009) predicted that an effect of market forces in dating markets is that highly desirable people will choose highly desirable partners, doing so because they can (they have access, and those partners are more desirable). Less desirable people must choose from less desirable people because they cannot access other options. It is possible to relate this concept to that of “liking reciprocity,” a mutual romantic interest between people; attraction or interest is less likely to be reciprocal where the two people concerned are of different market values. These ideas are supported by the literature and provide an alternative explanation for the “likes attract” idea; it only appears that likes attract because the market is forcing decisions that support the idea (Conroy-Beam et al., 2019; Wood & Brumbaugh, 2009). Despite this support, liking reciprocity remains an important but often overlooked component of partner selection literature (Günaydin et al., 2013).

Researchers have also developed different models of pathways to relationship formation, to complement partner selection theories (Günaydin et al., 2013; Levinger & Snoek, 1972). Levinger and Snoek’s (1972) model of pair relatedness is laid out in three stages: awareness, during which two people become aware of one another; surface contact, during which two people interact and learn about each other; and mutuality, during which two people have formed a romantic relationship. Stages are distinct in their level of interdependence, starting from nothing at awareness through to higher levels at mutuality. This model is useful as it delineates stages where various aspects of partner selection may take precedence (Eastwick et al., 2014). Researchers could use this model to target stages in their studies and this may provide alternative explanations for conflicting results; if past studies ignored stages, that oversight might account for apparently conflicting results (Eastwick et al., 2014).

In their chapter on “finding the one,” Günaydin et al. (2013) presented a process model of human partner selection. The model centres around what makes up a pool of partners and how this pool is reduced systematically until someone finds “the one”—the person or persons that are suitable long-term romantic partners for an individual. “Accessibility” is foremost in this model; if one cannot access a mate, they cannot be a potential partner for them. Accessibility can include, physical and social circle proximity, and cyber proximity (access via online means). “Appeal” is considered next; the individual reflects on which accessible potential partners are appealing and worthy of consideration. Third, mutual interest. It is proposed that a relationship would not be possible without “mutual interest.” This requirement for mutual interest further reduces the pool of potential partners, as not everyone that appeals to an individual, will in turn find that individual appealing (Wood & Brumbaugh, 2009). Finally, the experiences of the relationship determine whether those potential partners may or may not be “the one.” This model and Levinger and Snoek’s (1972) model are similar but can have unique applications in research, providing alternative ways of conceptualizing and studying partner selection.

Other models of partner selection, such as Li et al.’s (2002) mate preference priority model, focus on the decision-making in partner selection. Li et al. argue that individuals have minimum thresholds that must be met or exceeded for a potential partner to be considered. This process may be viewed in terms of Levinger and Snoek’s (1972) awareness or surface contact stages, or the Günaydin et al. (2013) appeal stage. Li et al. (2002) also borrowed from the economics literature with the idea of necessities and luxuries. They suggest that individuals will prioritize their preferences first in terms of partner characteristics they deem necessary (requiring minimum thresholds to be met), and then in terms of “luxuries.” These ideas have been supported in research (Li et al., 2013) and provide important insight into the arguably overlooked psychological processes of partner selection.

van Anders’ (2015) sexual configurations theory is a significant attempt to integrate some of the perspectives reviewed above. Sexual configurations theory looks to move beyond heteronormative and binary gender norms by introducing gender and sex sexualities. “Gender sexuality” describes how a person is attracted to gender(s), the psychological features of a person, or socially prescribed attributes of a gender. “Sex sexuality” describes how a person is attracted to physical sex characteristics (primary or secondary). Individuals possess a specificity in these sexualities, from highly specific (attracted towards a specific gender or set of sex characteristics) to non-specific (attracted to multiple genders or sex characteristics). Furthermore, van Anders suggests “separability,” in which someone may have distinct, separate, gender and sex sexualities, or a combined “gender/sex sexuality” that aligns more with binary gender norms (attraction to men/women appearing masculine/feminine, respectively, in appearance and behaviour). For example, if an individual does not see gender and sex as separate concepts, they are unlikely to have separate gender and sex sexualities. Sexual configurations theory taps into social constructs surrounding gender and sex which are rarely considered in the literature. The ideas also begin to consider the psychological processes of partner selection, again, a critical but under-researched aspect of partner selection.

Through sexual configurations theory, van Anders (2015) also challenges the simplified nature of reproductive fitness as characterised in the partner selection literature. Van Anders achieves this by reframing reproductive fitness into two evolution-driven concepts: “eroticism” and “nurturance.” Eroticism involves physically driven phenomena that may drive partner selection, such as arousal, lust, or reproduction. Nurturance involves psychologically driven phenomena, such as love, caregiving to partners and children, and intimacy. This perspective does not dismiss the idea that reproductive fitness could be a factor in partner selection, instead making it part of a broader picture. Despite their work being a good example of integrative theory, van Anders appears to overlook or dismiss some relevant perspectives. For example, the importance of resources a partner could provide is not directly addressed and interdependence theory is ignored, despite these factors perhaps fitting van Anders’ conceptualisation of nurturance.

### Summary

Theories within the partner selection field are numerous and it appears researchers are either resistant to adapting and integrating multiple theories, or the theories are difficult to reconcile (Conroy-Beam, 2021; van Anders, 2015). Arguably, evolutionary and social psychology researchers have pushed for their theories without much integration of alternatives (Li et al., 2015; Wood & Eagly, 2002). There are attempts to overcome these shortcomings by integrating theories (Fletcher et al., 1999), but these have generated other issues (Sparks et al., 2020; Valentine et al., 2019). Unfortunately, empirical testing of more recent attempts at integration (van Anders, 2015) are also a recent phenomenon (Schudson et al., 2017). Market theories are promising, but share a problem with process models, in that they may be too general and miss important nuance in the psychological processes of partner selection (Conroy-Beam et al., 2019; Eastwick et al., 2023). The field may also be failing to adapt to contemporary social changes in much of the West, such as a decline in desire for children (Weston et al., 2005) and a reduction in heteronormative ideals (Boxer, 2012). Despite these obstacles, the field of partner selection has made important progress (Buss, 1989; Eastwick et al., 2011; Fletcher et al., 1999), albeit that the complexity of partner selection phenomena remains difficult to address (Eastwick et al., 2023).

## Methodologies and Findings in Partner Selection Research

### The Ideal Standards Model

Fletcher et al. (1999) seminal “ideal standards model” has inspired the dominant methodology in the partner selection field over the last two decades (Burke, 2007; Csajbók & Berkics, 2017; Sparks et al., 2020). Fletcher et al. (2014) stated that selecting a suitable long-term romantic partner is an important decision which usually requires high levels of cognition. To inform the partner selection process, people draw on knowledge of themselves and of partners; they gain this knowledge from personal experience and from external sources, such as family and the media (Chen & Austin, 2016). Drawing on this knowledge, ideal standards regarding potential partners are formed. These ideal standards, or ideals, are then applied to potential partners to evaluate their suitability. A potential partner meeting an individual’s ideals could lead to a relationship, while failure to meet their ideals could cause hesitation or rejection. Alternatively, an individual may change their ideals to match a potential partner or their perception of a partner to match ideals, perhaps as a functional adaptation to preserve relationships or relationship satisfaction (Gerlach et al., 2019).

Building from the defining work of Buss and Barnes (1986) and Buss (1989), Fletcher et al. (1999) studied ideal preferences by examining partner trait preference dimensions using factor analysis in a New Zealand university student sample. The dimensions could then be used as the ideals to be measured in research; three dimensions emerged in their study. First, warmth-trustworthiness, which includes traits such as “understanding,” “supportive,” and “considerate.” Higher scores on warmth-trustworthiness indicate a preference for a warm, caring, and trustworthy partner, someone who could be relied upon for support and affection. Second, vitality-attractiveness, which includes traits such as “adventurous,” “nice body,” and “outgoing.” Higher scores on vitality-attractiveness indicate a preference for a physically attractive, active partner, someone who is confident, sexy, and outgoing. Last, status-resources, which includes traits such as having a “good job” and being “financially secure.” Higher scores on status-resources indicate a preference for a successful, well-provisioned partner, who shares value like religion and is of appropriate age.

The sample of the Fletcher et al. (1999) study was exclusively heterosexual and gender binary, a common limitation in the literature. There were no significant sex differences in ratings on the three dimensions of partner preferences. Across the sample, warmth-trustworthiness rated highest, followed by vitality-attractiveness, and status-resources rated lowest. These findings contradicted evolutionary predictions that men would favour vitality-attractiveness and women would favor status-resources. Notwithstanding this study, the search for sex differences in partner preference persists with conflicting results (Conley et al., 2011; Li et al., 2015).

### The Trait Preference Paradigm

The ideal standards model (Fletcher et al., 1999) set the paradigm for twenty-first century enquiry into long-term romantic partner selection with the first psychometrically validated preference scale; however, the application of the model is frequently more about trait preferences than an ideal-actual comparison. One such application is to investigate if alternative factor structures are more suitable. For example, Atari and Jamali (2016) recruited a sample of women in Iran and had them report their ideal partner preferences. Similar dimensions to the ideal standards model were observed: “kindness-dependability” (warmth-trustworthiness), “attractiveness-sexuality” (vitality-attractiveness), and “status-resources.” Two further factors were identified, “education-intelligence” and “religiosity-chastity.” Intelligence has been increasingly supported in the literature as a distinct dimension and as an important partner selection criterion (Csajbók & Berkics, 2017; Gignac et al., 2018). Shared values like religion and chastity have similar support but are usually only distinct in cultures where such values are strong aspects of individuals’ lives (Bejanyan et al., 2014; Hynie et al., 2006).

Cultural learning appears to create a perceived need for certain traits in a partner; simply put, an individual’s context impacts on their partner preferences (Guo et al., 2017). Marlowe (2004) highlighted the impact of context while reporting on the mate preferences of the Hadza people, who are hunter-gatherers in Tanzania. Hadza women preferred “foraging ability” and “intelligence” significantly more than Hadza men, who had some preference for foraging ability, but little preference for intelligence. Marlowe argued that in their society, these traits would translate to Fletcher et al. (1999) status-resources dimension. This argument suggests men are perceived as needing these skills to collect and secure resources for their families, supporting evolutionary predictions about the preferences of heterosexual women.

Marlowe (2004) also found that Hadza men preferred the fertility dimension significantly more than women, which is consistent with evolutionary predictions that men are focused on health and potential indicators of reproductive fitness. However, the fertility dimension includes traits of “can have kids”, “will have kids”, and importantly “once had kids” and “(has) lots of kids.” This finding appears to be more easily explained by cultural perspectives than evolutionary. If caring for children already, the women would arguably have depleted resources to care for the man’s future children. Desiring a woman with children may contravene ideas of chastity or some religious ideals where the belief is that women should only have children to one man, within marriage (Atari & Jamali, 2016); a cultural difference already identified in Buss’ (1989) cross-cultural study. Marlowe’s (2004) findings highlight the unlikelihood of evolution or culture explaining trait preferences in isolation and the need to consider context, individual differences, and the combination of multiple factors and perspectives.

Studies inspired by economic theories have adopted a mate budget design to investigate whether trait preferences change under different budgetary conditions (Jonason et al., 2017; Li et al., 2002; Thomas et al., 2019). For example, Li et al. provided participants with a list of traits and a budget of “mate dollars” to build a partner based on traits purchased. One mate dollar corresponded to a 10% increase in one trait. Participants were allocated to either a low, medium, or high budget condition, with $20, $40, and $60, respectively. This approach tests the mate preference priority model (minimum thresholds on traits must be met to proceed), while also investigating which traits are necessities and which are luxuries. Li et al. found that, for women, necessities were yearly income and intelligence (spent first and to a threshold, then stopped spending), while creativity was a luxury (spent after necessities were met). For men, necessities were physical attractiveness and intelligence, while creativity and special non-work talents were luxuries; it is unclear in the original and subsequent papers using the measure what “special non-work talents” means to participants (Edlund & Sagarin, 2010). These findings and other cross-cultural budget studies (Thomas et al., 2019) support evolutionary predictions about sex differences in partner preferences. However, it should be noted that Thomas et al. (2019) found moderate significant differences in budget allocation between Eastern and Western cultures, for all traits they measured. This finding continues to highlight the impact of cultural learning and context on trait preferences.

The distinction between short-term and long-term partners is an important context that can influence trait preference and partner selection (Li et al., 2015). At first, short-term mate preferences were believed to be almost exclusively reliant upon physical attractiveness (Wiederman & Dubois, 1998). However, evidence is emerging that while physical attractiveness is important, people also prefer a warm and intelligent short-term partner (Jonason et al., 2013; Regan et al., 2000; van Straaten et al., 2007). There are a few potential explanations for inconsistent findings in this area (Li & Kendrick, 2006), but the most relevant to this review is whether people can accurately report preferences for long-term partners (Li et al., 2015). Li et al. speculate that even when studies clearly label questions as regarding long-term romantic partners, it may not be enough to overcome a participant’s short-term mindset or context; they accordingly report preferences for short-term relationships, not long-term. To address this problem, Li et al. suggest that recruitment be limited to only those interested in long-term romantic relationships. They also suggest that studies should consider age and more comprehensive trait lists.

Csajbók and Berkics (2017) perceived the primary focus on ideal preferences to be a problematic issue in the literature. To overcome this, they also measured participants’ self-perceived ratings on the same trait list they used to report ideal preferences. Csajbók and Berkics thought that analysing preferences and self-ratings together within factor analysis would provide a more accurate set of dimensions. Their rigorous factor analysis extracted seven factors, “warmth,” “stability,” “appearance,” “passion,” “status,” “intellect,” and “dominance.” Again, the dimensions of Fletcher et al. (1999) are supported with dimensions of warmth, appearance, and status. Intellect or intelligence is supported as a distinct dimension, as it was in Atari and Jamali (2016). Stability seems to be a dimension that would fit within warmth-trustworthiness, but was distinct here, and dominance a dimension somewhat unique to this study. It appears that while sometimes the Fletcher et al. (1999) dimensions are relevant and useful, there may be further dimensions that warrant investigation in different samples and contexts.

### Making Predictions Using Trait Preferences

Researchers have also been interested in the predictive utility of trait preferences (Campbell & Stanton, 2014; Eastwick et al., 2014; Fletcher et al., 2014) —what constructs and outcomes can trait preferences predict? Csajbók and Berkics (2017) attempted to use traits to predict self-perceived mate value, an individual’s perception of their worth as a potential mate. They used participants’ self-ratings on the seven dimensions of partner traits they extracted as predictors. For men, appearance was a strong significant predictor, with passion, status, intellect, and dominance all significant predictors to a lesser degree. For women, appearance was also a strong significant predictor, with only passion and dominance as weaker significant predictors. Overall, 45–54% of self-perceived mate value was significantly accounted for by the participants’ self-ratings on the seven trait dimensions, indicating that traits played a substantial role in individuals’ self-perception in partner selection contexts. Mate value is an important construct that recent research has found to have an impact on people’s partner selection behaviour and other aspects of functioning (Bosson et al., 2022; Charlot et al., 2020; Edlund & Sagarin, 2010; Williams & Sulikowski, 2020).

Predicting whom people are likely to choose as a long-term romantic partner is arguably the most important prediction one could make with trait preferences data (Campbell & Stanton, 2014). Fletcher et al. (2014) conducted a study to test the predictive utility of trait preferences in live interaction scenarios. Participants pre-reported their trait preferences four to 10 days before having a 10-min interaction with a stranger. Vitality-attractiveness, the dimension regarding physical attractiveness and confident, outgoing traits, was the most powerful dimension for predicting romantic interest. These findings were the same for men and women, with women reporting generally lower romantic interest; this difference in romantic interest seemed to be due to women perceiving less vitality-attractiveness in partners and potential partners failing to meet their minimum standards of vitality-attractiveness. Li et al. (2013) similarly supported the predictive utility of trait preferences, finding that a higher preference for social status and physical attractiveness influenced partner selection decisions.

The predictive utility of trait preferences is not always supported, as Eastwick et al. (2014) observed after conducting a meta-analysis of predictive trait preference literature. They found that sex differences in predictors of romantic interest were non-significant. For men and women, physical attractiveness was the best predictor of romantic interest, followed by earning prospects. Eastwick et al. also found that trait preferences were good at predicting romantic interest towards hypothetical partners but not after they had met potential partners face-to-face. They speculate that this is due people being poor at predicting their emotions generally, including romantic affect. Campbell and Stanton (2014) directly contest these claims in their review of the Eastwick et al. (2014) meta-analysis studies. They argue that none of the studies directly assessed relationship formation, and thus, any claims about poor predictive validity are weak. This debate remains open in the literature and could be a productive path forward to address the complexity of the partner selection process (Eastwick et al., 2023).

### Summary

The ideal standards model has influenced partner selection research for over two decades and established a paradigm where trait preferences are the focus of most studies. Trait preference research seems to be associated with evolutionary perspectives, which may limit the potential for other traits, preferences, or influences to be studied. Whether researchers choose to employ the Fletcher three-dimensional model or observe their own factors, inconsistencies in approach and findings make it hard to draw conclusions. Nowhere is this more evident than in the use of trait preferences to predict romantic interest and partner selection. When trait preferences fail to consistently predict these outcomes, it leads to questions about the current applications of the ideal standards model. Perhaps trait preferences are useful in some areas and applications, but not others; this remains an unsettled issue (Eastwick et al., 2023).

## Current Approaches, Findings, and Open Questions

### Explicit and Implicit Processing in Partner Selection

One potential explanation for trait preferences predicting romantic interest in hypothetical partners, but not in face-to-face potential partners, is explicit versus implicit processing (Eastwick et al., 2014). Eastwick et al. (2011) investigated this idea by measuring both explicit and implicit preferences for physical attractiveness. When responding to photographs, explicit preference significantly predicted romantic interest; but this was only the case for men responding to photographs of women, especially when they reported a higher preference for physical attractiveness. Implicit preference did not significantly predict romantic interest when responding to photographs. Interestingly, Eastwick et al. suggest that this sex difference arises because reviewing photographs is an unusual way to select partners. Eastwick and their team published their study one year before popular photograph-based dating app “Tinder” was released; photograph-based dating apps quickly became popular and subsequently garnered academic attention (Hess, 2014).

Eastwick et al. (2011) conducted another study where the participants’ response target was a live confederate whose attractiveness was manipulated with clothing and make-up. This manipulation was effective, as participants’ ratings of the confederate’s attractiveness ranged from 1.2 to 7.8 on a 9-point Likert scale. Explicit preference did not significantly predict romantic interest in the live confederate, but implicit preference did—for men and women. Eastwick et al. claim that when people review photographs or descriptions of potential partners, it is an abstract process. These abstract evaluations are similar in nature to reporting explicit preferences; thus, explicit preferences are predictive when reviewing photographs and accompanying descriptions. Conversely, meeting someone face-to-face is a live, in-the-moment process. This leads to more affective evaluations, which implicit preferences are better suited to represent. Thus, implicit preferences have predictive utility for face-to-face meetings. Implicit processes appear to be important in partner selection, but in the decade since the study by Eastwick et al. (2011), there has been little focus on the subject (Eastwick et al., 2023).

### Measuring Partner Preferences Indirectly

While Eastwick et al. (2011) measured implicit preference with an implicit association task (Go/No-go Association Task; Nosek & Banaji, 2001), Wood and Brumbaugh (2009) developed a different approach, “revealed preferences.” This approach is not a traditionally “implicit” one, but rather a way to indirectly measure an individual’s trait preferences via their attraction towards potential partners. A five-person team rated 98 photographs on various traits, resulting in each photograph having an average rating for each trait; this was a valid and reliable method in their study, and in others (South Palomares & Young, 2017). Participants then viewed each photograph and rated how attracted they were to the photograph subject: the “target.” Revealed preferences were then calculated based on the correlation between the research team’s trait ratings and each participant’s reported attraction towards the targets. A positive correlation considering the participant’s ratings across all photographs would indicate a general preference for that trait. For example, if a participant’s attraction was generally higher towards targets with higher intelligence ratings, the resulting positive correlation would reveal the participant’s preference for intelligence. The opposite would be true for negative correlations, which would indicate the trait was undesirable to that participant.

Wood and Brumbaugh (2009) conducted factor analyses using the revealed preferences in their general online sample of heterosexual, gay, and lesbian participants; they found two factors. First, the alpha factor which included “confidence” and “being sexually suggestive”, with “toned” and “masculine” for male targets, and “curvaceous” and “feminine” for female targets. Second, the communal factor which included “intelligence” and being “conventional,” “soft-hearted,” and “classy”. Traits not loading onto any factor included, being “well-groomed,” “trendy,” “thin,” and whether the target was “smiling.” Wood and Brumbaugh used reliability measurements as a proxy for whether a trait was consistently desired. For men, thin and trendy were consistently desired; for women, masculine, toned, and thin were consistently desired; for all participants, being formal and smiling were consistently not desired. These factors could be seen as implicit dimensions of trait preferences, which could be a key to improved predictive utility.

As Wood and Brumbaugh (2009) collected data on who the participants were and were not attracted to, they investigated whether there was consensus on which targets were attractive. Men, both heterosexual and gay, had higher consensus on attractiveness of targets than women did, both heterosexual and lesbian. Wood and Brumbaugh speculate that this may be due to men having stricter criteria for attractiveness than women. The higher consensus on who is attractive could also lead to more difficulty in securing a desirable partner, due to increased competition as more people agree on who is desirable. Men preferred traits of classy, well-groomed, trendy, thin, confident, seductive, and intelligent, whereas women preferred traits of, smiling, sensitive, and unconventional. These differences were similar if comparing gay and lesbian samples, indicating that general trait preferences may be driven more by gender differences than by sexual identity.

Participants in Wood and Brumbaugh (2009) were also asked about their dating interest in the photograph targets. Overall, there was less dating interest than there was attraction, with a medium effect size. When comparing attraction to dating interest, traits in the alpha factor reduced in importance with effect sizes ranging from small to medium. The importance of communal traits increased, albeit with small effect sizes, indicating that these traits were more valued in potential romantic partners. There was slightly less consensus in dating interest than there was for attraction. The findings of Wood and Brumbaugh highlight the importance of novel approaches and provide fertile ground for future research. However, the revealed preferences method of measurement has been questioned in recent reviews (Eastwick et al., 2018).

### Computer Modelling and Machine Learning of Trait Preference and Partner Selection

#### Computer Modelling

Recently, researchers have been turning to computer modelling and machine learning to test the predictive validity of trait preferences (Conroy-Beam et al., 2019; Conroy-Beam, 2021; Eastwick et al., 2023; Joel et al., 2020). Conroy-Beam et al. (2019) set the stage for their computer modelling study with the following proposition: the complexity and imperfection of potential partners necessitates a psychological system that consolidates and integrates information about the individual’s preferences, to ultimately perceive the mate value of potential partners. They adopted a computer model to approximate this process using five dimensions: kindness, intelligence, and health (each consistently desired across cultures), alongside physical attractiveness and financial prospects (each somewhat consistently ranked differently by sex). Data were collected from participants regarding their preferences for, and self-ratings on, these dimensions. Computer modelling then produces “offspring,” which then produce further offspring, iterating or “evolving” over time. Evolved offspring are then compared to the original data to see if the computer model approximates reality. Conroy-Beam and their team was successful in creating a computer model that approximated their original data set; this indicates that the dimensions and processes may sufficiently represent reality.

Conroy-Beam et al. (2019) were interested in “preference distance”: the difference between an observer’s preferences and a target’s self-ratings on the five dimensions measured. A participant or digital offspring who had a low average preference distance was indicative of someone with generally high mate value. The computer model found that people with high mate values had higher trait preferences, and therefore selected high mate value partners. This finding could support “market forces” theories of partner selection, wherein high mate value individuals are desirable and have the option of high mate value partners as result. Conroy-Beam et al. suggest that this model highlights the integrative psychology of partner selection, but also acknowledge that this is a complex process. This complexity implies that there is a high level of effort required to calculate a potential partner’s preference distance. As a result, this process may not be functional in evolutionary terms—which may suggest that evolutionary factors, while important, cannot alone explain this specific process, or partner selection generally—although Conroy-Beam et al. did not suggest this. Conroy-Beam et al. also acknowledge that it is not an individual alone that is involved in the partner selection process, but also external parties such as friends and family, highlighting the need for further research. These types of acknowledgements are common throughout the literature but seem to be rarely integrated into subsequent research.

Conroy-Beam (2021) and Conroy-Beam et al. (2022) extended computer modelling of partner selection by undertaking “Couple Simulation.” This process uses data from real romantic couples to test the capacity of various models to recreate those same couples. Both studies had some success, with the best models recreating approximately 50% of couples. This is an impressive result given how complex partner selection choices are, as evidenced in this review. Conroy-Beam (2021) found the most useful model of partner selection to be the resource allocation model. This is a novel model that frames partner choice as series of resource decisions that are first based on mate value and then based on the degree of mutual resource investment between two individuals. Conroy-Beam suggests that this makes sense, as people likely have surface level interest in many partners but mostly pursue those who have mutual interest—for example, those who would agree to a date. Counterexamples exist, such as individuals pursuing uninterested parties, but these do not take away from the utility of the model which makes a strong contribution to our understanding of partner choice.

Conroy-Beam et al. (2022) took the findings supporting the resource allocation model and aimed to investigate ways that preference and perception could interact, which they termed “partner evaluation.” Three partner evaluation models were tested. First, weighted sum, where the mate value is the sum of a partner’s trait values altered by the individual’s preference importance. Second, aspiration threshold, where the individual’s highest ranked trait preference is used to eliminate partners low on that trait. The process is then repeated for the next highest ranked trait until one partner remains or the process would eliminate all partners. Third, preference distance, where mate value is calculated based on how well the partner’s traits match the individual’s ideals, with closer matches having less distance, and therefore, more value. Each computer modelled individual is assigned resources which are then reassigned according to the resource allocation model. When each of the partner evaluation models were applied to the same data set, preference distance emerged as the strongest model. This finding supports the notion that ideals are a primary driver of partner selection, a foundational idea of the Ideal Standards Model (Fletcher et al., 1999). Conroy-Beam et al. even suggest that variables other than ideals could be responsible for limitations on predictive power throughout the literature.

The findings of couple simulation computer modelling studies (Conroy-Beam, 2021; Conroy-Beam et al., 2022) are important and provide many opportunities for further research. Conroy-Beam et al. detail some limitations in their study; for example, models performing worse with newer relationship data (< 5 years together). Individual differences in selection psychology could also influence results, such as individuals only applying ideals for certain preferences, in specific situations, or not at all. It would be interesting to probe whether individuals can conceptualise their own selection psychology, perhaps drawing on the partner evaluation models used by Conroy-Beam et al., and then apply couple simulation with the model that matches the sample’s self-identified psychology.

In Conroy-Beam et al. (2022), the slightly more successful model of preference distance allowed for partners to be selected despite severely undesirable traits; for example, a highly attractive person with sadistic tendencies having equal preference distance to a somewhat attractive person who is slightly grumpy. Conroy-Beam et al. believe this does not make sense in theory, but it could. If physical attractiveness is a proximal selection metric, as with first impressions during dating, it could lead to a scenario where that information gets the person with detrimental traits into a relationship, alike perhaps to the foot-in-the-door concept (Freedman & Fraser, 1966; Sundie et al., 2012). Once a relationship has begun based on an attractiveness ideal, the undesirable traits could be ignored or minimized due to any number of reasons, from interdependence (Thibaut & Kelley, 1959), to love (Burke, 2007), or sunk cost influences (Hou et al., 2022). This undesirable trait scenario could also be addressed with ideas like the model of paired relatedness (Levinger & Snoek, 1972). It may be that these unfavourable outcomes occur due to quick but resource-intensive progress through relatedness stages. Researchers should not only consider pathways to successful outcomes, but also pathways to unsuccessful outcomes, or successful outcomes with detrimental aspects.

#### Machine Learning

A machine learning study by Eastwick et al. (2023) attempted to predict romantic partner choices, with a focus on the early phases of relationship development. The dataset was based on 208 single participants, who reported on potential romantic partners at multiple timepoints, leading to 1065 unique potential partners and 7179 reports over seven months. This large dataset was then used within a “random forests” form of machine learning. This process involved thousands of dataset subsamples which were used to test predictor validity for various outcomes including romantic interest. Predictors found to be consistently valid across subsamples were retained. These retained predictors are then entered into models together and only predictors that remained valid across combined models were retained. Consequently, a set of robust predictors emerge, which can be examined without the need to manually eliminate redundant predictors.

Eastwick et al. (2023) investigated the predictive utility of two sets of predictors: first, target-specific constructs, involving variables related to one potential partner; and second, individual differences, involving measurements that were related to participants, such as their personality. Few individual differences were significant as predictors and target-specific constructs accounted for more variance in romantic interest beyond individual differences. Adding individual differences to a model with target-specific constructs reduced the variance accounted for; this may be due to individual differences only adding noise to the data and diminishing potentially useful predictors. Target-specific constructs that were substantial predictors include attractive, exciting, and attachment-based variables such as desire to seek proximity, distress at separation, and seeing someone as a secure base. Romantic interest peaked at first report, before dropping a whole scale point (out of 7) on average at second report, three weeks later. This decline was gentler but still pronounced even if a relationship began with the target. Target-specific constructs could predict romantic interest at first report (peak) but had no predictive utility at final report and could not predict changes in romantic interest over time. Although this study involved hundreds of predictor variables, prediction of relationship formation and romantic interest over time was not possible with any model.

A secondary goal of Eastwick et al. (2023) was to test the predictive utility of ideal partner preferences. A preference for attractiveness was the most powerful predictor of romantic interest; other substantial predictors included exciting, creative, supportive, and confident. However, there were no significant interaction effects between any combination of ideal trait preference and the level of that same trait in a potential partner. This finding indicates that trait preferences may be useful in predicting romantic interest, but similarities between an individual’s ideals and a potential partner’s actual traits appear to have no predictive value. Importantly, it was the participant’s own report that determined the actual trait variable; there were no significant interactions between their abstract ideals and their report on the same traits in a potential partner. This finding goes against the core prediction of the ideal standards model (Fletcher et al., 1999) and suggests that trait preferences may not be a relevant factor in partner selection—at least in the early stages of relationship development.

One promising theoretical direction discussed by Eastwick et al. (2023) is dyadic communication. This type of communication could be key in early relationship development. Significant predictors of romantic interest within this area include perceived interest, self-disclosure, and mixed signals. When discussing mixed signals, Eastwick et al. cite Tennov (1979) who argues that romantic infatuation is a mix of hope and uncertainty. Tennov’s argument sums up the complex and confusing nature of partner selection processes for those involved, and for academics studying the phenomenon. Researchers acknowledge this complexity and address it through increasingly complicated versions of established approaches. Findings emerging from these approaches further our understanding by testing well-established theories and providing crucial information about the complexity of partner selection. However, reviewing these findings together reveals mixed results, suggesting a need for new approaches that could further address complexity.

### Summary

There appears to be a distinct difference in how people select partners in abstract contexts (such as reviewing photographs and descriptions) versus live contexts (such as a first date). Attempting to probe implicit preferences and processing could reveal valuable information about the partner selection process. Advanced computational methods have enabled more nuanced work within the trait preference paradigm. This development has resulted in some intriguing findings about predictors of romantic interest and partner selection. However, that list of predictors is far from settled and trait preferences may be only a minor predictor. Arguably, researchers can no longer just acknowledge the complexity of partner selection processes; instead, established approaches should be adapted with complexity in mind, which will likely require the development of new ideas and approaches.

## Issues in the Partner Selection Field

### Theoretical Issues

The partner selection literature is expansive, with countless perspectives, claims, and counterclaims, which increases the likelihood of unresolved theoretical issues (Eastwick et al., 2018; Fletcher et al., 2020). Perhaps the most significant of these is the dearth of theoretical flexibility, and the associated problem of poor theoretical integration—the latter despite innovations in the field. Notwithstanding being generally sound research, Conroy-Beam et al. (2019) exemplifies this situation. Conroy-Beam et al. used an innovative approach and highlight the need for people to integrate information about potential partners to evaluate their mate value. While recognizing a complex integrative process, Conroy-Beam et al. generally fail to integrate theoretical perspectives, offering a mostly evolutionary perspective. They write:

However, mate selection’s proximity to reproduction means, in natural selection’s eyes, it is among the most important decisions an organism will ever make. Especially in human long-term mating, where romantic partners have a variety of avenues to influence one another’s reproduction, the benefits of scrutinizing partners on more dimensions might outweigh the costs of added decision-making complexity (Conroy-Beam et al., 2019, p. 8).

Leaning on reproductive fitness and evolutionary perspectives alone may no longer be sufficient (van Anders, 2015; Weston et al., 2005). To their credit, Conroy-Beam et al. (2019) do consider heuristics within the same paragraph. However, this discussion could be strengthened by some social psychological theories regarding the origins and impacts of heuristics; for example, Burke’s (2007) work on falling in love as a partner selection heuristic. Instead, Conroy-Beam et al. adhere to a relatively strict evolutionary perspective, and integration is only touched upon, despite recognition that the process under scrutiny is integrative.

Even integrative theories, such as van Anders’ (2015) sexual configuration theory, can be critiqued based on theoretical limitations. When discussing their concept of “eroticism” (the arousal, lust, and reproduction construct), van Anders advises against conceptualising this construct as multi-faceted. Specifically, they state that viewing sexuality as part physical and part emotional is not as useful as their concepts of eroticism and nurturance. Eroticism could be multi-faceted, part physical or physiological, and part emotional or psychological. These ideas are supported in the literature (Althof, 2012; Brody & Costa, 2017) and ignoring them is arguably unhelpful. Furthermore, sex differences may come in to play, as women appear to have more negative psychological experiences after casual sexual encounters, indicating a distinct psychological component to physical intimacy (Wesche et al., 2018). Sexual configurations theory is an otherwise inclusive and progressive work, which makes this type of limitation more apparent and worthy of attention.

### Trait Preference Issues

Many studies on romantic partner selection follow Fletcher et al. (1999) method of recording individuals’ ideal trait preferences and, occasionally, their self-ratings on traits (Csajbók & Berkics, 2017). Li et al. (2015) reviewed challenges in trait measurement as potential impediments to observed sex difference in mate preferences. They suggest that age could play a part in sex differences going unobserved, as women over the age of 35 may find factors around reproductive fitness less important than younger women. Again, an evolutionary bias should be flagged; desire for reproduction or raising children would need to be qualified in the data as some may not have this desire (Pralat, 2020). Li et al. also call for a wider range of traits in studies, which has been supported in the literature that opted to measure fewer traits (Conroy-Beam et al., 2019). Finally, objective measures of the important physical attractiveness and social status traits are called for. Subjective ratings of physical attractiveness and social status could be confounding variables, even if people seem to be good judges of attractiveness levels generally (Wood & Brumbaugh, 2009). Other traits that deserve more attention include sociosexuality, communication, and attachment styles (Eastwick et al., 2023). Overall, which traits are selected and how those are measured should be considered with more scrutiny (Eastwick et al., 2011; Wood & Brumbaugh, 2009).

The trait preference paradigm of the early twenty-first century has generated interest in the partner selection field (Eastwick et al., 2014; Fletcher et al., 1999; Li et al., 2002). However, the strong focus on trait preference has overshadowed a key component of the ideal standards model that was responsible for initiating this line of research. Fletcher et al. (1999) state that reviewing and selecting partners requires high levels of cognition, which underpins a psychologically complex process that has been underexamined for two decades. After the limits of trait preference research became apparent, studies have turned to psychological processes as a focus (Conroy-Beam et al., 2022; Eastwick et al., 2023). Some attempts have been made to investigate psychological processes of partner selection, but these attempts are based on marriage as an outcome and are broad in focus (French & Kus, 2008; Xie et al., 2015). There is a need to integrate current and future findings into thinking around the psychological processes of partner selection. Furthermore, it may be useful to examine what members of the public understand the process of partner selection to involve (Sprecher et al., 2008).

### State of the Partner Selection Field

After a half-century of partner selection research, seminal works such as Buss (1989) and Fletcher et al. (1999) have initiated a renaissance in the field. Recent work in this area began with relatively uncomplicated ideas about dimensions of preference and ways in which people may evaluate ideals versus actualities in potential partners. Over the next two decades, difficulties have begun to arise as inconsistent results make conclusions difficult. Within the literature, writers contest the best ways to approach the field and debate which theories are most useful. Emerging from this debate are recent findings that may set the stage for a new renaissance in the field. However, the opportunities inherent in newer work may not be seized due to recurrent issues of strict theoretical adherence and a lack of integration. If future research is to progress the field, it may be argued that there is a need to integrate current knowledge with new findings, methods, and theories, while also looking to better understand the psychological processes of partner selection.

## Suggestions for Next Steps in the Partner Selection Field

### Qualitative Research

Perhaps the most pressing need in the partner selection research field is the need for more qualitative research (Chen et al., 2015; Sprecher et al., 2008). Sprecher et al. and Chen et al. (2015) are rare examples of the literature calling for more qualitative research in the area. The literature such as Campbell and Fletcher (2015) and Conroy-Beam et al. (2019) identify concerns that could potentially be addressed by qualitative enquiry but do not acknowledge this potential. Of the few partner selection-related qualitative studies found during this review, most focus on somewhat specific populations, for example, Iranian women (Shahrabi Farahani et al., 2019; 2020), young Mormon women (Stacy, 2004), or cisgender partners of transgender people (Forde, 2011). To better understand the psychological process of partner selection, a more general focus on the lived experiences of people in the general population is needed.

Qualitative research could enable direct scrutiny of the processes people are experiencing (Silverman, 2020) when selecting romantic partners. This potentially contrasts with quantitative approaches in the field which rarely address these processes and instead incorporate an increasing number of variables framed within complex statistical models (Conroy-Beam et al., 2019; Eastwick et al., 2023). Qualitative enquiry into how people experience selection of romantic partners could generate novel findings, and perhaps new variables or ideas to be applied within quantitative or mixed method approaches (Braun & Clarke, 2013; Creswell & Plano Clark, 2017). Selection of a romantic partner is often an important experience and therefore might be richly recalled by participants, making it suitable for qualitative data collection and analysis (Smith et al., 2009). Long-term romantic partner selection is a near universal phenomenon, so phenomenological methods such as interpretative phenomenological analysis (Smith et al., 2009) may be a sound starting point.

#### Diversity in Partner Selection Research

It may be apparent that most of the studies reviewed included exclusively heterosexual samples (Fletcher et al., 1999; Li et al., 2013). Kalakewich (2018), Wood and Brumbaugh (2009), and Wu et al. (2019) are examples of studies that include non-heterosexual samples. Kalakewich (2018) and Wood and Brumbaugh (2009) both observed no differences in trait preferences based on sexual identity. This lack of difference may indicate that further research is not necessary. However, Wu et al. (2019) reported findings with a bisexual sample that highlighted areas where differences may be present and relevant to the specific population being studied. If future research were to observe no differences across diverse experiences, this would be helpful in building our understanding of partner selection generally. If, alternatively, future research was to observe differences across diverse experiences, this would be helpful in building our understanding of aspects specific to certain populations. Each set of findings would be important, and both could contribute towards our knowledge of partner selection phenomena. Seemingly contradictory observations could simply be a manifestation of a complexity in partner selection that researchers should acknowledge.

People who identify as asexual are underrepresented in the partner selection literature and research involving this population could deepen our understanding in the field (Maxwell, 2017). Maxwell’s qualitative findings highlight important differences in experience and preference for people identifying as asexual. For example, the need for asexual individuals to negotiate the socially dominant need for sexual relations and attraction, a consistent focus of evolutionary perspectives (Fletcher et al., 1999; van Anders, 2015), or asexual individuals’ overwhelming primary preference for intelligence. People who identify as asexual have a unique experience in partner selection; attraction, an assumed core component of selection, is not experienced in the same way. Therefore, there is a need for research to understand this experience and apply that understanding to the broader picture of human partner selection.

Trans and gender diverse people are also underrepresented in the literature and have unique experiences that research could investigate (Richards, 2016). The intersection of gender and sexuality, including sexual identity, impacts heavily on trans and gender diverse people’s lives (Richards, 2016). Much of the partner selection research in this area has been regarding cisgender people’s views on trans and gender diverse partners (Blair & Hoskin, 2018; Forde, 2011). Gender has been speculated to have a large impact on sexuality (van Anders, 2015). Therefore, it is arguably important to study the trans and gender diverse population who are perhaps best suited to comment on the impact of gender (Richards, 2016). Future research needs to include this population and incorporate relevant theories, like queer theory (Forde, 2011). It is important for the developing partner selection literature that researchers collaborate with the trans and gender diverse community on the impact of gender in partner selection, and on partner selection research generally.

### Online Dating and Dating Apps

Online dating has become a major feature in modern partner selection, especially with the popularity of recent dating apps (Hess, 2014; Bruch & Newman, 2018). Research in this area unfortunately carries the same flaws as the broader literature; studies focus on trait preference (Hitsch et al., 2010) or specific populations (Gavin et al., 2019; Shakouri & Shafiyi, 2015). Currently, earlier “face-to-face” findings have been replicated in online dating samples, but as the two lines of research share flaws, this replication is of arguably limited significance. As with the broader literature, there needs to be more focus on the psychological processes of partner selection when using online dating platforms. Furthermore, scrutiny of differences and similarities in the psychological processes between face-to-face and online environments could be illuminating. The COVID-19 pandemic has seen an increase in online dating and has potentially impacted partner selection behaviour (Alexopoulos et al., 2021; Goldstein & Flicker, 2020), an interesting and fertile area for important future research.

### Mate Evaluation Theory

Eastwick et al. (2023) machine learning study provides robust evidence against the idea that partner selection choices can be predicted using an individual’s preferences and their evaluations of targets. Eastwick et al. propose in their “mate evaluation theory” that this is because there are two components in partner selection, a “feature lens” and a “target-specific lens.” The feature lens involves individual-specific factors, such trait preferences, similarity preferences, or mate value perceptions. The target-specific lens involves intra-relationship factors that are unique to the person-to-person interaction, such as shared experiences, knowledge of reciprocal liking, and inside jokes. The target-specific or intra-relationship factors were reported to be better predictors of romantic interest in Eastwick et al. (2023). Current research focuses more on individual-specific factors and, as a result, is limited in its ability to consistently predict partner selection choices, even when accounting for both individuals in a dyad. Future research will need to account for the target-specific lens, to properly examine those cases where these intra-relationship factors are driving partner selection. This is a promising area of future research and qualitative studies may be the key to uncovering the nature and themes of these intra-relationship factors.

### Clarifying Levels of Analysis

Partner selection researchers may be failing to acknowledge the level of analysis they are using in their research designs and reports, leading to some of the issues covered in this review. Pietraszewski and Wertz (2022) describe the levels of analysis researchers use while studying the mind: intentional, functional, and implementational. The intentional level describes mental phenomena as personal, involving feelings, beliefs, decision-making, and evaluation. The functional level describes phenomena as mechanisms involving systems that automatically integrate information without executive or personal oversight. The implementation level describes phenomena as physical events involving hormonal or neurochemical changes. Researchers failing to acknowledge these levels when comparing findings, ideas, or theories may see them as being in conflict when the reality may be one of complementarity. For example, social psychological perspectives like cultural influence (Chen & Austin, 2016), evolutionary perspectives like sex differences (Li & Meltzer, 2015), and biological perspectives like hormonal influence (Han et al., 2020) may, respectively, suit intentional, functional, and implementational levels of analysis. These perspectives do not need to compete or conflict but can instead complement one another.

Pietraszewski and Wertz’s (2022) arguments about levels of analysis being complementary lend themselves to addressing the lack of integration detailed in this review. Earlier, we made the point that ignoring stages of relationship formation (Günaydin et al., 2013; Levinger & Snoek, 1972) may be the cause of conflicting results. The same could be happening due to unclarified levels of analysis; that is, results appear to conflict because they emerge from differing levels of analysis. Furthermore, it is possible that the levels of analysis could be present and useful to acknowledge within other models, such as the stages of relationship formation. Applying this thought could lead to conceptualizations of partner selection phenomena that, while necessarily complex, enhance our understanding. Indeed, a different review to the one currently presented could examine partner selection literature through the lens of levels of analysis, stages of relationship formation, or both simultaneously.

### Integrative Framework

Integration is key but limited within in the partner selection literature. van Anders’ (2015) sexual configurations theory and Finkel and Eastwick’s (2015) focus on goals over rewards (their ‘instrumentality principle’) are both good examples of concepts that begin integrative work. Alongside levels of analysis awareness, an integrative framework may help advance these ideas further, perhaps like the impact of the biopsychosocial model in the biomedical field (Bolton & Gillett, 2019). Explanations for influences on partner selection could be delineated through an integrative framework. For example, one might consider a biopsychosocial perspective on reproduction; reproduction could be a biological drive that requires fulfillment, or a psychological need to raise children for the future, or a social requirement that children are produced for the good of the family or community. Importantly, it could be any combination of these explanations or others. Delineation in this manner does not necessitate separability or superiority. Instead, delineation helps highlight influences that may or may not interact inside a complex system and assists in understanding potential sources of, and impacts on, those influences.

### Psychological Processes and Prediction

The partner selection literature has a major focus on the prediction of an individual’s choice of long-term romantic partner. This focus on prediction may sometimes hinder novel thinking about the psychology of partner selection, which usually manifests in the processes behind selection choices. Researchers might incorporate increasingly specific variables to predict choice, from dyadic experiences (Eastwick et al., 2023) to preference distance (Conroy-Beam et al., 2022), but may nevertheless overlook the psychological process and processing of these choices. Conroy-Beam et al. (2022) used prediction to gain insight into the processes of partner selection which greatly contributed to our knowledge within this field. However, it is possible individual differences in those processes are being neglected through a focus on the typical individual. The predictive utility of various models may be improved if differences in processing are acknowledged and perhaps integrated into research designs. Overall, it is important to understand not only whom people choose, but also the psychological processes, experiences, and individual differences involved in their choices.

### Summary

There is a need for new ideas within partner selection research that move beyond the current paradigm. Currently, the focus is on the prediction of outcomes, while the psychology behind these outcomes is neglected. Qualitative research appears to be an underutilized approach that could generate ideas and specifically investigate the psychological process of partner selection. Research should also consider populations beyond heterosexual and cisgender groups, and ideas beyond the hetero- and cis-normative. Sexual identities or patterns such as asexuality and polyamory, and gender identities such as trans and non-binary, could provide unique insight into the partner selection process. Researchers should move away from unitary theoretical approaches and attempt to be integrative in terms of theory to begin capturing the complexity of partner selection phenomena. These suggestions are not solutions to problems within the literature, but instead are focused on potentially important steps that may contribute to the health and success of the field.

## Conclusion

This review has highlighted prominent theories, perspectives, methodologies, and findings in the literature regarding the selection of long-term romantic partners. Issues in the literature include strict adherence to singular perspectives and a lack of integration thereof, which partially manifests in a failure to integrate new findings and alternative methods into established knowledge. Furthermore, there is difficulty in accurately measuring preferences and predicting romantic partner selection choices. Overall, these problems may be due to scholarship in this area acknowledging, but not sufficiently addressing, the complexity of the partner selection process. Recent findings have begun to provide the tools needed to start effectively addressing that complexity. Perhaps paramount in addressing the complexity of the partner selection process is a need to focus on what psychological processes are behind it. The content of this review and accompanying suggestions will hopefully contribute towards the continuing efforts to understand why and how people select long-term romantic partners. It is hoped that this knowledge can help people to understand themselves and others throughout the life-transforming and sometimes stressful experience of selecting a long-term romantic partner.
